# Translation, cross-cultural adaption, validity and reliability of a composite physical function scale for adults aged 65 + years in a Danish context

**DOI:** 10.1186/s12877-023-04240-2

**Published:** 2023-08-29

**Authors:** Bettina Mølri Knudsen, Birgitte Nørgaard, Hanne Rasmussen, Inge H. Bruun

**Affiliations:** 1 Lillebaelt Hospital - University Hospital of Southern Denmark, Sygehusvej 24, 6000 Kolding, Denmark; 2 Institute of Regional Health Research, University of Southern Denmark, J.B. Winsløws Vej 19, 5230 Odense M, Denmark; 3 Lillebaelt Hospital - University Hospital of Southern Denmark, Beriderbakken 4, DK-7100 Vejle, Denmark; 4 Department of Public Health, University of Southern Denmark, J.B. Winsløws Vej 9B, 5230 Odense M, Denmark; 5 Department of Physiotherapy and Occupational Therapy, Slagelse Hospital, Ingemannsvej 18, 4200 Slagelse, Denmark; 6 Department of Physiotherapy and Occupational Therapy, Lillebaelt Hospital, Sygehusvej 24, 6000 Kolding, Denmark

**Keywords:** Ageing, Composite physical function scale, Functional ability, Cognitive interviews, Danish translation, Assessment

## Abstract

**Background:**

To prevent or postpone dependence on help in everyday activities, early identification of adults aged 65 + years at risk of functional decline or with progressing functional decline is essential. The American Composite Physical Function (CPF) scale was developed to detect and prevent this age-conditioned decline. In this study, the aim was to translate and adapt the scale into a Danish version and assess the validity and reliability in Danish adults aged 65 + years.

**Methods:**

A forward–backward translation procedure was used, followed by an expert panel review to finalise the Danish version of the CPF scale. In the subsequent pre-test, three-step cognitive interviews and hypotheses testing were performed to evaluate the validity, and a test–retest was done to assess reliability.

**Results:**

In the pre-test, 47 adults participated in three-step cognitive interviews, and 45 adults answered an online version of the scale. In terms of content validity, the scale was relevant and easy to answer, although many informants skipped the instruction to the questionnaire, which may negatively impact face validity. Construct validity showed a significant difference in CPF scores in adults aged 65 + years by residence and activity level and a decreasing CPF score with increasing age. The reliability test showed an excellent kappa (0.92).

**Conclusion:**

The scale covering daily activities helps to identify adults aged 65 + years with reduced physical functions or at risk of loss of independence. Further research is needed to assess the CPF predictive value for adults aged 65 + years at risk of or with a progressing physical decline.

**Supplementary Information:**

The online version contains supplementary material available at 10.1186/s12877-023-04240-2.

## Background

For the majority of adults aged 65 + years, maintaining physical independence in activities of daily living (ADL) is the most important health outcome [1]. Sickness and ageing are two reasons for endangering this independence, causing an unmet or additional need for help in ADL [2–4]. The age-conditioned decline entails loss of muscle mass followed by reduced physical performance and functional decline and, consequently, the risk of dependence or need for help in ADL [2, 5–7]. A significant association has been demonstrated between resistance exercises and upper and lower body strength improvement, indicating that resistance exercises can be the key to maintaining independence or at least postponing dependence [8–11]. Despite this knowledge, adults aged 65 + years in need of rehabilitation are often not identified until there is an immediate need for help in daily activities. Earlier identification is therefore needed to prevent or postpone dependence [12, 13].

Physically, adults aged 65 + years often experience restrained instrumental activities of daily living (IADL), such as difficulties in housekeeping and shopping before they experience restrained basic activities of daily living (BADL) [7]. The latter includes activities such as dressing and grooming. The need is typically identified in the healthcare system using a questionnaire referring primarily to BADL [12, 13]. An easily accessible scale covering both BADL and IADL is expected to contribute to earlier identification and enable the initiation of targeted resistance exercises when the adults aged 65 + years still have the physical energy for it.

The Composite Physical Function (CPF) scale, developed in the US, meets this requirement with its 12-item hierarchical scale that assesses physical function from BADL to IADL and more advanced activities such as strenuous sports/exercise activities [14]. To use the CPF scale in a Danish healthcare setting, translation into Danish and assessment of validity and reliability among adults aged 65 + years are needed. Thus, the aim of this study is to translate and culturally adapt the CPF scale to a Danish context and assess the validity and reliability of the scale.

## Methods

### Design

Using a cross-sectional design, translation and adaptation were planned and executed using the guideline by Beaton et al., including the following five stages: translation, synthesis, back translation, expert committee review, pre-testing and the additional, optional testing of the adapted version [15]. COSMIN (COnsensus-based Standards for the selection of health Measurement INstruments) psychometric guidelines [16] were used to assess the validity and reliability of the adapted version in a test re-test. The translation process was evaluated and reported using the COSMIN reporting guidelines for patient-reported outcome measurement instruments [17].

### Informants and recruitment

Participants aged 65 + years were recruited from June to October 2021. To ensure variability in gender, living arrangements, residence, and the ability to participate in strenuous physical activity, informants were recruited by addressing them personally in different settings (group A) or by using snowball sampling (group B). The different settings for recruitment of group A include: hospital visits to participate in a rehabilitation program, visits to a private fitness centre for training, or they were approached in the pre-test coordinator’s local environment. To avoid a selected group, the participants had no knowledge of the visit by the researcher. In addition, we included adults aged 65 + years from three different nursing homes in the area. Here, the staff identified participants based on their mental state who were included if they were willing to participate. Group B was recruited from the researchers’ network by email, and those who accepted inclusion were asked to forward the adapted version for testing to adults aged 65 + years in their network.

### The Composite Physical Function (CPF) scale

The 12-item CPF scale covering BADL (for example, dressing or bathing yourself), IADL (for example, household chores and shopping) and more advanced activities (for example, strenuous sports, heavy household chores, and exercise activities) was an extension of the five and/or six-item scales by Siu, Reuben, and Hays, the four-item scale by Rosow-Breslau, and additional three items from the National Health Interview Survey (in the following collectively referred to as the original CPF scale—see Supplementary material 1) [14]. The original CPF scale was developed to assess physical ability in adults aged 60–94 in the Senior Fitness Test [14], based on the theoretical background that physical decline, whether due to disease or inactivity, is predominantly modifiable through activity interventions [18].

The scores of the original CPF are 2 (“can do)”, 1 (“can do with difficulty or with help”), or 0 (“can not do”), respectively, resulting in total scores ranging from 0–24, with a calculated sum score of 24 indicating full function and a score of 0 indicating that the individual is unable to perform any of the activities.

The original CPF was validated in three ways [14]; 1) convergent validity by determining its correlation with the abovementioned scales by Siu, Ruben, Hays and Rosow-Breslau; 2) criterion validity using a treadmill performance, and 3) discriminant validity by looking at the sensitivity of the test identifying hierarchical levels of functional ability. Convergent validity showed a high correlation (0.92 < r < 96). Criterion validity showed a moderate correlation between CPF and treadmill (*r* = 0.69). Significant differences were found in discriminant validity between the treadmill scores and advanced functional ability (CPF score = 24 points) and intermediate functional ability (CPF score = 18–23) [14].

In the assessment of the validity in the original CPF, information on physical activity was dichotomised into 1) high-active adults aged 65 + years, defined as those participating in strenuous activity three or more times per week, and 2) low-active adults aged 65 + years as those not participating in strenuous activity or being active on an irregular basis (≤ 2 times a week). Regarding reliability, a test–retest demonstrated a 0.94 correlation measured 2 to 4 weeks apart [14].

### Translation process – stage I-IV

At stage I, the original CPF was forward translated by two bilingual translators with Danish as their native language with different backgrounds (one is a physical therapist, and the other is a linguist). The two forward translators worked independently during the translation process. The initial translations were compared and merged into a single version by the pre-test coordinator at stage II. Any discrepancies between the versions were analysed and resolved by the two translators and the pre-test coordinator.

At stage III, the back-translation was carried out by two native English speakers: one was a physical therapist and the other with an academic background. After the back translations, at stage IV, an expert committee reached a consensus on any discrepancies and reviewed all versions. The expert committee included all translators, a methodologist, a clinician, a physical therapist, and the pre-test coordinator. The translation process resulted in a Danish version of the CPF consistent with the original version, except for minor adjustments mainly concerning units of measuring not applied in a Danish context. The final version, including reports from each stage, was sent to the developers of the original version, who had accepted the translation into a Danish version.

### Pre-testing – stage V

At the final stage of the adaption process, the pre-test was performed. According to Beaton et al., 30–40 respondents are recommended [15]. In group A, the informants were asked to fill out an information sheet with demographic data. Then, the informants were asked to complete a paper-based version of the CPF scale in a three-step cognitive interview process, including 1) think aloud, 2) probes, and 3) debriefing [15, 19]. Finally, the informants were asked to indicate their confidence in understanding the question correctly for each item and rate these on a scale from 0–10, 0 being very uncertain that they understood the question correctly and 10 being absolutely sure. The informants were asked to complete the questionnaire again after 7–14 days using either an online or a paper-based version.

Group B was also asked to fill out the information sheet and CPF scale, but as they answered an online version, they did not participate in the three-step cognitive interview process. As part of the online version, group B was, however, able to give feedback and make suggestions to improve the questionnaire in an open text field. Any feedback was reviewed and analysed along with the input from the cognitive interviews as part of the content validity assessment.

### Reliability – adapted version

According to Beaton et al., it is recommended to do further testing of the adapted version to assess the reliability [15]. This assessment may be done as part of the pre-testing process (stage V), but with the use of a larger sample size, an absolute minimum of 50 is recommended by COSMIN [16]. No significant changes were made to the Danish CPF scale in stages I-V. Thus, the reliability assessment in the test–retest was done in continuation of stage V, considering the recommendations for sample size.

### Assessing validity

#### Validity was assessed using

- 1) content validity, including face validity, defined as the degree to which the content of a measurement scale adequately reflects the construct to be measured [20]. In this study, this was done by using cognitive interviews.
- 2) convergent validity indicating that two measures believed to reflect the same underlying phenomenon will correlate highly. As this was tested in the original version, it is also added to this study [21].
- 3) construct validity defined as the degree to which the scores of a measurement scale are consistent with hypotheses [20].

Assessment of content validity including face validity is recommended by Beaton. Convergent and construct validity assessment is an optional test of the adapted version [16].

The hypotheses testing of construct validity using a priori hypotheses related to differences in the scores between subgroups. We tested the following hypotheses:

1. Comparing adults aged 65 + years living in a nursing home with adults aged 65 + years living in their own homes, we expected a significant difference in the mean CPF scores between the groups, with a greater functional decline among adults aged 65 + years living in a nursing home.
2. With regard to physical activity, we expected a significant difference in the CPF score when comparing the high-active adults aged 65 + years with the low-active adults aged 65 + years.
3. Due to the association between age and physical ability, we expected a decreasing CPF score with increasing age. Moreover, based on the hierarchical levels, we expected that the age-related decrease in the total CPF scores was caused by difficulties performing the advanced activities followed by difficulties in IADL.

### Assessing reliability

We tested reliability, defined as the extent to which scores for informants whose situation was unchanged, were the same for repeated measurement over time [20]. According to COSMIN, a minimum of 60 participants is required to assess reliability [16]. This was done by doing a test–retest of the CPF scale with an interval of 7–14 days. This interval was chosen 1) to secure a time frame short enough to minimise the risk of progressing physical change in the informants and 2) to secure a time frame long enough to ensure that the informants were no longer able to recall their previous answers.

### Data analysis

To examine content validity, data from the cognitive interviews were analysed under the following four themes: comprehension, retrieval, judgment, and response [22].

The convergent validity was tested the same way as in the original version, thus with the scales by Siu, Ruben, Hays and Rosow-Breslau, using Spearman’s rank correlation coefficient.

The a priori hypotheses were tested using the Mann–Whitney-U-test since the total sum score was not normally distributed.

We considered the distance between the three response options as unequal, as the extent of difficulties doing an activity seems to be greater if you go from ‘can do with difficulty’ or ‘with help’ to’cannot at all’ compared with going from ‘can do’ to ‘can do with difficulty or with help’. Therefore, the assessment of reliability in the test–retest was done by using the quadratic weighted kappa [23]. A value below 0.40 is considered poor or fair/slight, depending on Fleiss or Landis and Koch. A value below 0.75 is fair to good or moderate/substantial, and a value above 0.75 is considered excellent according to Fleiss or above 0.8 is almost perfect by Landis & Koch [16]. The software STATA was used for the data analysis.

## Result

**Fig. 1:**
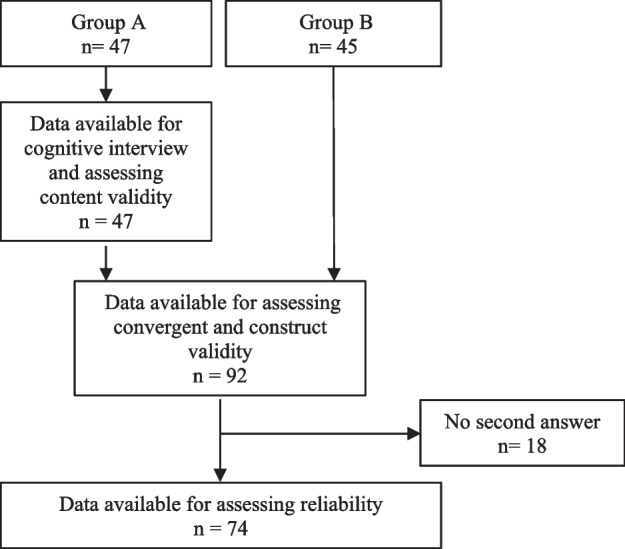
Flowchart of the recruiting process

A total of 92 adults aged 65 + years accepted inclusion; 47 were recruited for group A and thus included in the three-step cognitive interviews. Additional 45 informants were recruited in group B. The recruitment process is visualised in Fig. 1.

In Table 1, we present the characteristics of the informants by group. Only informants recruited for group A participated in the cognitive interviews. Both groups participated in convergent and construct validity assessment, and adults aged 65 + years with a second response were part of the test–retest.

**Table 1:** Characteristics of the informants (*n* = 92)

	Group A		Group B	
	(*n* = 47)		(*n* = 45)	
Characteristic	median	IQR^1^	median	IQR
Age (years)	76	(70–84)	71	(68–72)
	n	%	n	%
Gender				
Women (*n* = 57)	31	66	26	67
Living arrangement				
Alone	21	45	5	11
Cohabitation	26	55	40	89
Residence				
House/apartment	31	66	45	100
Nursing home	16	34		
Using walking devices or a wheelchair				
All the time	12	25	0	0
Sometimes	6	13	3	7
Not at all	29	62	42	93
Participating in strenuous physical activities				
No	13	28	11	24
Yes	34	72	34	76
	median	IQR	median	IQR
Times per week	3	(2–3)	3	(3–4)
Total sum score CPF	23	(14–24)	24	(23–24)

^1^ IQR: interquartile range

The mean time for answering the questionnaire was 2.67 min, standard deviation (SD) of 2.4 measured in group A, *n* = 47.

### Content validity

Below, the results from the three-step cognitive interviews are presented in the four themes:

1. Comprehension
  None of the items was considered irrelevant or understood differently from the intention. However, many respondents did not read the initial instructions to the CPF scale explaining that the responses should indicate *their ability* to perform the activities, not if *they actually do* them. Skipping the instructions might negatively impact face validity as the subsequent test battery may seem irrelevant to the respondent if it includes activities that they actually do not perform.
2. Retrieval
  None of the informants found it difficult to remember their ability to perform the activities. However, most of those living in nursing homes had not performed some of the activities in many years, such as doing light or hard household chores. The informants residing in their own homes or residential homes were clear about their ability to perform or not perform the activities.
3. Judgement
  Some informants questioned the items on lifting and carrying 10 or 25 lbs as it is not specified for *how long* the weight must be carried. Thus, they needed further details or accuracy to respond to the items satisfactorily. A few informants found it difficult to respond to the item on shopping for groceries or clothes as it is two very different activities, making it difficult to respond if the informant can shop for one but not the other.
4. Response
  The informants depending on the use of a walking aid tended to answer the items within walking distance without using their walking aid (can do). To them, using a walking aid was considered normal and not categorised as something they ‘can only do with help’. Some informants did not associate ‘help’ with a physical aid such as a walking aid but associated it with personal help.

Overall, the informants found the questionnaire easy to answer, and they were confident that they understood the questions correctly.

In terms of content validity, the informants found the questions relevant because the items reflected their everyday life. Overall, they found that the items, to some extent, covered their physical functional ability. The distribution of the informants’ responses during the interviews is available in the Supplementary material 2.

### Convergent validity

The convergent validity was tested the same way as in the original version, thus with the scales by Siu, Ruben, Hays and Rosow-Breslau using Spearman’s rank correlation coefficient. In the original version of the CPF scale, the correlation was (0.92 < r < 96). The correlations (r) in this present study were 0.85 < r < 0.91.

### Construct validity

As hypothesised, a significant difference in the CPF sum score was found between adults aged 65 + years living in their own homes with a median of 24 (IQR 23–24) and those living in a nursing home with a medium of 9 (IQR 4–15) (discriminant validity) (*P* < 0.001).

**Fig. 2:**
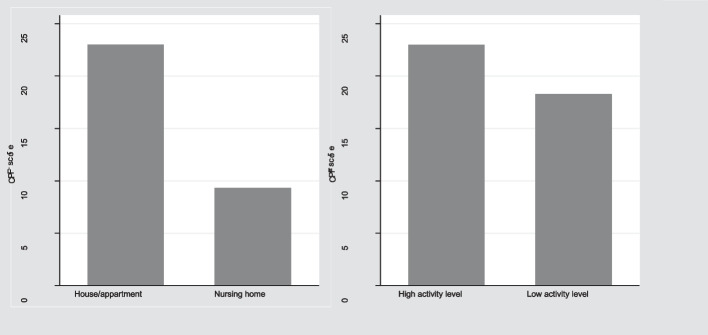
The CPF scores on adults aged 65 + years by residence or activity level

A significant difference was found when comparing the adults’ physical activity levels. The median CPF sum score for the high-active adults was 24 (IQR 23–24) and for the low-active adults 23 (14–24)(*P* < 0.001) (Fig. 2).

**Fig. 3:**
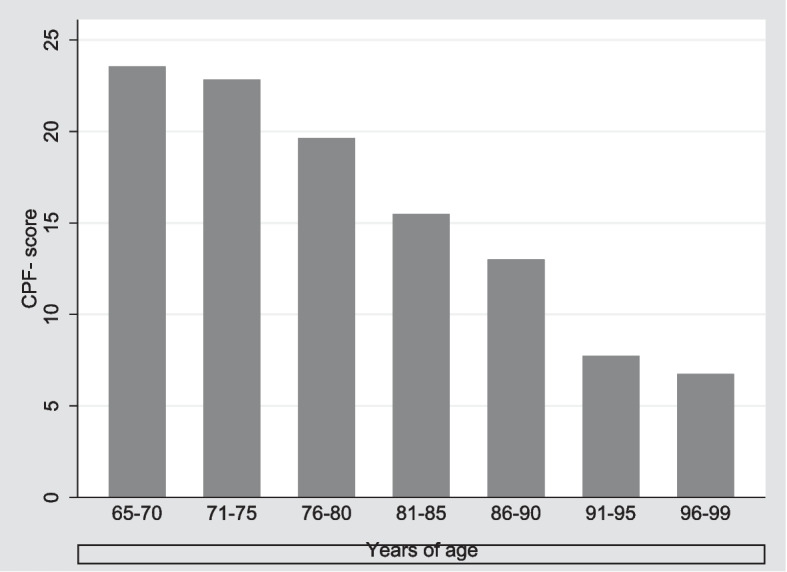
The CPF scores on adults aged 65 + years by age

The CPF score of adults aged 65 + years according to age is displayed in Fig. 3, showing that the CPF score declines with age.

As to the association between age and physical ability, we expected a decreasing CPF score with increasing age. Moreover, based on the hierarchical levels, we expected that the age-related decrease in the total CPF scores was caused by difficulties in performing advanced activities followed by difficulties in IADL. Table 2 shows that the decline in the CPF score for adults aged 81–85 relates to advanced activities.

**Table 2:** The percentage of adults aged 65 + years who can do the activity

Years of age	65–70		71–75		76–80		81–85		86–90		91–95		96–99	
	*n* = 33		*n* = 28		*n* = 17		*n* = 4		*n* = 2		*n* = 4		*n* = 4	
Activity	n	%	n	%	n	%	n	%	n	%	n	%	n	%
Take care of own personal needs – like dressing yourself	33	100	28	100	15	88	2	50	2	100	2	50	2	50
Bathe yourself, using tub or shower	33	100	28	100	14	82	2	50	2	100	1	25	2	50
Climb up and down a flight of stairs (like to a second story in a house)	32	97	28	100	13	76	2	50	1	50	1	25	1	25
Walk outside (one or two blocks)	32	97	28	100	15	88	3	75	1	50	2	50	1	25
Do light household chores – like cooking, dusting, washing dishes, sweeping a walkway	33	100	28	100	15	88	3	75	1	50	1	25	0	0
Shop for groceries or clothes	32	97	27	96	13	76	2	50	0	0	0	0	0	0
Walk ½ mile (6–7 blocks)	32	97	26	92	14	82	2	50	1	50	0	0	0	0
Walk 1 mile (12–14 blocks)	31	93	25	89	13	76	1	25	1	50	0	0	0	0
Lift and carry 10 lb (full bag of groceries)	33	100	26	93	12	71	2	50	0	0	1	25	2	50
Lift and carry 25 lb (medium to large suitcase)	30	91	20	71	9	53	1	25	0	0	0	0	0	0
Do heavy household activities – like scrubbing floors, vacuuming, raking leaves	32	97	26	93	14	82	0	0	0	0	0	0	1	25
Do strenuous activities – like hiking, digging in the garden, moving heavy objects, bicycling, aerobic dance activities, strenuous calisthenics, etc	30	92	23	82	10	59	0	0	0	0	0	0	0	0

### Reliability

The second questionnaire (the test re-test) was answered on average eleven days (SD 3.69) after the first answer. The kappa for the total CPF score was 0.9290, corresponding to an excellent kappa by Fleiss [16]. The correlation between the CPF in the first and second tests was *r* = 0.9377 (*p* =  < 0.001). The kappa values for each item, which was fair to good, are displayed in Table 3 [16].

**Table 3:** T quadratic weighted kappa for each item within the CPF scale

**Questionnaire**	Agreement	Expected agreement	Kappa	*P* value
Take care of own personal needs – like dressing yourself	98.0	95.6	0.5404	< .001
Bathe yourself, using tub or shower	97.6	93.6	0.6326	< .001
Climb up and down flight of stairs (like to a second story in a house)	94.6	84.0	0.6633	< .001
Walk outside (one or two blocks)	97.0	87.6	0.7553	< .001
Do light household chores – like cooking, dusting, washing dishes, sweeping a walkway	95.3	87.4	0.6260	< .001
Shop for groceries or clothes	97.6	81.2	0.8742	< .001
Walk ½ mile (6–7 blocks)	94.9	76.3	0.7861	< .001
Walk 1 mile (12–14 blocks)	96.3	71.6	0.8691	< .001
Lift and carry 10 lb (full bag of groceries)	98.7	73.9	0.9482	< .001
Lift and carry 25 lb (medium to large suitcase)	93.3	68.6	0.9462	< .001
Do heavy household activities – like scrubbing floors, vacuuming, raking leaves	97.6	74.1	0.9088	< .001
Do strenuous activities – like hiking, digging in garden, moving heavy objects, bicycling, aerobic dance activities, and strenuous calisthenics etc	96.6	65.1	0.9031	< .001

## Discussion

In this study, the CPF scale was successfully translated and adapted into Danish. To our knowledge, the original CPF scale has only been translated into Spanish for use among the Chilean elderly [24]. The results of this study align well with our findings as all items were clear and understandable [24]. In addition, an American study used the original CPF to test the validity and stability of the scale among women with fibromyalgia. The study showed acceptable concurrent validity and stability and a valid scale for assessing physical abilities among women with fibromyalgia [25].

The content validity in this study was tested in adults aged 65 + years recruited from different settings. The mix of settings was necessary to test the hypotheses between subgroups at the hierarchical level and to investigate the purpose of the CPF scale: to identify adults aged 65 + years at risk of progressing in physical decline. To test the hypotheses, the study population must consist of adults aged 65 + years at risk of or with a progressing physical decline. Hence, we had to recruit respondents from different settings. The results of the content validity, including face validity, also showed that comments to the items primarily came from informants with physical and/or functional decline, making it untenable to exclude this subgroup from the study population.

The content validity showed some limitations. Many informants did not read the instructions before filling out the CPF scale. If they skip the instructions, the informants may feel that the questions are irrelevant. The instructions should therefore be clearly presented to the informants ensuring that they answer all items based on their ability to perform the activities and not if they actually do them. The cognitive interviews also revealed that some informants questioned the definitions of the items, making it difficult to provide a correct answer. Also, the informants depending on the use of a walking aid tended to answer that they performed the activity without help. However, this is considered a general limitation of the CPF scale and not specifically related to the Danish version. In the Danish version, we have therefore added instructions to the informants to guide them in how to respond when using walking aids or a wheelchair (Supplementary material 1).

Concerning the construct validity using hypotheses testing, results displayed that the CPR score differs for community-residing adults and adults aged 65 + years living in a nursing home. Moreover, the CPF scores follow the ageing process, a process that entails loss of muscle mass, followed by reduced physical performance and functional decline [7, 26]. Although the result is expected, based on the well-known ageing process, it is essential to note that it is based on only 14 adults in the last four age groups [7]. Results indicate that the scale is relevant for identifying adults aged 65 + years and, by this, identifying adults in need of targeted resistance exercise. The latter is an important intervention to achieve maximum muscle strength and functional performance [9, 10, 27]. However, further research is required to assess the CPF predictive value for adults aged 65 + years at risk of or with a progressing physical decline, especially since the CPF was developed for functional fitness normative scores and clinically relevant fitness standards for maintaining physical independence in later years [28, 29].

The hypotheses relate to the ageing process of adults aged 65 + years, making the CPF a measurement of age in this study. However, although loss of muscle mass in terms of sarcopenia is mainly age-related, sarcopenia also occurs earlier in life [30]. Furthermore, the muscle mass in adults aged 65 + years also varies depending on the adults’ lifelong level of activity and type of activity [31]. Therefore, age alone is insufficient to identify adults aged 65 + years with functional decline.

The sample size is a limitation in the assessment of validity. Only a few participants aged 81 and above showed a decreased median CPF sum score. This decline was as hypothesised, but due to the low number of participants, we cannot make valid conclusions based on this. Moreover, as demonstrated, the majority of adults in this study can take care of their own personal needs, involving a potential risk of ceiling effects.

This study’s results are exclusively based on the adults’ responses, and we have no proof that the answer is correct. A simultaneous test of the respondent’s physical ability using physical performance measurements, such as a hand grip strength or chair stand test, would have validated the response of the adults and strengthened the study.

We tested the reliability using a test–retest and demonstrated an excellent kappa, implying a good correlation between the first and second assessment of the informants, either using a paper-based version of the scale or/and an online version. Using both a paper and online version imposes limitations on the reliability and validity of the data obtained. Even though the online questionnaire is an adaption of a traditional paper-based version, more is needed to assume that the psychometric properties of the original version apply to the translated online version [32].

## Conclusion

The American CPF scale was translated and culturally adapted to a Danish setting. The translation and adapting process showed that CPF is easy to fill in and covers ordinary activities in daily life. The validity assessment demonstrated a scale with a hierarchical level corresponding to the well-known ageing process and a scale with excellent reliability. Testing of the scale in a Danish setting showed limitations regarding judgement of some items and how to perceive the response category on the ability to do the activities with help. The scale’s ease of use makes the Danish version valuable in measuring the physical function of adults aged 65 + years.

### Supplementary Information

**Additional file 1:** **Supplementary table 1.** Original CPF scale and final Danish version.

**Additional file 2:** **Supplementary table 2.** Distribution of responses from the pretest.

## Data Availability

The datasets used and/or analysed during the current study are available from the corresponding author on reasonable request.

## Abbreviations

ADL: Activities of daily living
BADL: Basic activities of daily living
COSMIN: Consensus-based standards for the selection of health measurement instrument
CPF: Composite Physical Function
IADL: Instrumental activities of daily living
IQR: Interquartile range
lbs: Pounds
SD: Standard deviation

## References

[1] Fried TR, Tinetti ME, Iannone L, O’Leary JR, Towle V, Van Ness PH. Health outcome prioritization as a tool for decision making among older persons with multiple chronic conditions. Arch Intern Med. 2011;171(20):1854–6. 10.1001/archinternmed.2011.424 PMC4036681 21949032

[2] Goldspink DF (2005). Ageing and activity: their effects on the functional reserve capacities of the heart and vascular smooth and skeletal muscles. Ergonomics.

[3] Gill TM, Allore HG, Gahbauer EA, Murphy TE (2015). The role of intervening illnesses and injuries in prolonging the disabling process. J Am Geriatr Soc.

[4] Boyd CM, Landefeld CS, Counsell SR, Palmer RM, Fortinsky RH, Kresevic D, Burant C, Covinsky KE (2008). Recovery of activities of daily living in older adults after hospitalization for acute medical illness. J Am Geriatr Soc.

[5] Young A (1997). Ageing and physiological functions. Philos Trans R Soc Lond B Biol Sci.

[6] Reid KF, Fielding RA (2012). Skeletal muscle power: a critical determinant of physical functioning in older adults. Exerc Sport Sci Rev.

[7] Elisabetta S, Stefano V, Giovanni Z, Jack MG (2014). Assessment of mobility status and risk of mobility disability in older persons. Curr Pharm Des.

[8] Chodzko-Zajko WJ, Proctor DN, Fiatarone Singh MA, Minson CT, Nigg CR, Salem GJ, Skinner JS (2009). American College of sports medicine position stand. Exercise and physical activity for older adults. Med Sci Sports Exerc.

[9] Peterson MD, Rhea MR, Sen A, Gordon PM (2010). Resistance exercise for muscular strength in older adults: a meta-analysis. Ageing Res Rev.

[10] Stewart VH, Saunders DH, Greig CA (2014). Responsiveness of muscle size and strength to physical training in very elderly people: a systematic review. Scand J Med Sci Sports.

[11] Liu CJ, Latham N (2011). Can progressive resistance strength training reduce physical disability in older adults? A meta-analysis study. Disabil Rehabil.

[12] Graf C. Functional decline in hospitalized older adults: it’s often a consequence of hospitalization, but it doesn’t have to be. American J Nurs. 2006;106(1):58–68, 52p. 10.1097/00000446-200601000-00032 16481783

[13] Hickman LD, Phillips JL, Newton PJ, Halcomb EJ, Al Abed N, Davidson PM (2015). Multidisciplinary team interventions to optimise health outcomes for older people in acute care settings: a systematic review. Arch Gerontol Geriatr.

[14] Rikli RE, Jones CJ (1998). The reliability and validity of a 6-minute walk test as a measure of physical endurance in older adults. J Aging Phys Act.

[15] Beaton DE, Bombardier C, Guillemin F, Ferraz MB (2000). Guidelines for the process of cross-cultural adaptation of self-report measures. Spine (Phila Pa 1976).

[16] de Vet HCW, Terwee CB, Mokkink LB, Knol DL (2011). Measurement in Medicine.

[17] Gagnier JJ, Lai J, Mokkink LB, Terwee CB (2021). COSMIN reporting guideline for studies on measurement properties of patient-reported outcome measures. Qual Life Res.

[18] Re R (1997). Assessing physical performance in independent older adults: Issues and guidelines. J Aging Phys Activity.

[19] Hak T, Van der Veer K, Jansen H (2008). The Three-Step Test-Interview (TSTI): An observation-based method for pretesting self-completion questionnaires. Survey Res Methods.

[20] Mokkink LB, Terwee CB, Patrick DL, Alonso J, Stratford PW, Knol DL, Bouter LM, de Vet HC (2010). The COSMIN study reached international consensus on taxonomy, terminology, and definitions of measurement properties for health-related patient-reported outcomes. J Clin Epidemiol.

[21] Portney LG, Watkins MP (2009). Foundations of Clinical Research.

[22] Collins D (2003). Pretesting survey instruments: an overview of cognitive methods. Qual Life Res.

[23] Cohen J (1968). Weighted kappa: nominal scale agreement with provision for scaled disagreement or partial credit. Psychol Bull.

[24] Merellano-Navarro E, Lapierre M, Garcia-Rubio J, Gusi N, Collado-Mateo D, Olivares PR (2015). Translation and cultural adaptation of the composite physical function for its use in chile. Rev Med Chil.

[25] Jones CJ, Rutledge D, Lindemann J, Rigali R: Validity and stability of the Composite Physical Functional (CPF) Scale for women with fibromyalgia. In: Proceedings of the National Fibromyalgia Association Fibromyalgia CME Conference: 2006. 17–19.

[26] Cruz-Jentoft AJ, Sayer AA (2019). Sarcopenia. The Lancet.

[27] Fiatarone MA, O’Neill EF, Ryan ND, Clements KM, Solares GR, Nelson ME, Roberts SB, Kehayias JJ, Lipsitz LA, Evans WJ. Exercise training and nutritional supplementation for physical frailty in very elderly people. N Engl J Med. 1994;330(25):1769–75. 10.1056/NEJM199406233302501 8190152

[28] Rikli RE, Jones CJ (2013). Development and validation of criterion-referenced clinically relevant fitness standards for maintaining physical independence in later years. Gerontologist.

[29] Rikli RE, Jones CJ (1999). Functional fitness normative scores for community- residing older adults, ages 60–94. J Aging Phys Act.

[30] Cruz-Jentoft AJ, Bahat G, Bauer J, Boirie Y, Bruyère O, Cederholm T, Cooper C, Landi F, Rolland Y, Sayer AA (2019). Sarcopenia: revised European consensus on definition and diagnosis. Age Ageing.

[31] Klitgaard H, Mantoni M, Schiaffino S, Ausoni S, Gorza L, Laurent-Winter C, Schnohr P, Saltin B (1990). Function, morphology and protein expression of ageing skeletal muscle: a cross-sectional study of elderly men with different training backgrounds. Acta Physiol Scand.

[32] Buchanan T (2003). Internet-based questionnaire assessment: appropriate use in clinical contexts. Cogn Behav Ther.

[33] Union. EPaCoE: The General Data Protection Regulations (GDPR). Regulation (EU) 2016/679. In., vol. 2016. EU: Official Journal of the European Union.; 2016.

[34] Committee Act 2020 (Section 14, paragraph 2). Retrieved from https://www.retsinformation.dk/eli/lta/2020/1338#P14. In.

