# Supplementary material for: Translation, cross-cultural adaption, validity and reliability of a composite physical function scale for adults aged 65 + years in a Danish context
Source: BMC Geriatr. 2023 Aug 29;23:526. doi: 10.1186/s12877-023-04240-2 (PMC10466833; doi:10.1186/s12877-023-04240-2)
Supplement: Supplementary file 1 — Additional file 1: Supplementary table 1. Original CPF scale and final Danish version. [file 12877_2023_4240_MOESM1_ESM.docx]

**Supplementary material 1**

*Supplementary table 1: Original CPF scale and final Danish version*

| **Original Version Item** | **Dansk version** |
| --- | --- |
| A Composite Physical Function (CPF) Scale | Kombineret fysisk funktionsskala |
| *Instructions: Please indicate your ability to do each of the following. (Your response should indicate whether you are able to do these activities, not if you actually do the activities):* | *Vejledning: Angiv venligst din evne til at udføre følgende aktiviteter. Dit svar skal vise, hvorvidt du er i stand til at udføre aktiviteten, ikke om du rent faktisk gør det:* |
| Take care of own personal needs – like dressing yourself ^a^ | Varetage personlig pleje, så som at tage tøj på |
| Bathe yourself, using tub or shower ^a^ | Gå i bruse eller karbad. |
| Climb up and down flight of stairs (like to a second story in a house)^b^ | Gå op og ned ad trapper, svarende til én etage |
| Walk outside (one or two blocks) | Gå 100 – 200 meter udenfor (svarende til cirka 5 minutters gang) *^1^ *se vejledning nedenfor* |
| Do light household chores – like cooking, dusting, washing dishes, sweeping a walkway | Udføre lettere huslige gøremål, så som at lave mad, støve af, vaske op eller feje |
| Shop for groceries or clothes ^a, b^ | Tage på indkøb efter dagligvarer eller tøj |
| Walk ½ mile (6-7 blocks) ^a, b^ | Gå cirka 1 kilometer (svarende til cirka 20 minutters gang) **^1^ se vejledning nedenfor* |
| Walk 1 mile (12-14 blocks)^c^ | Gå cirka 2 kilometer (svarende til cirka 45 minutters gang) *^1^ *se vejledning nedenfor* |
| Lift and carry 10 lb (full bag of groceries) ^c^ | Løfte og bære 5 kg, svarende til en fyldt indkøbspose |
| Lift and carry 25 lb (medium to large suitcase) ^c^ | ^c^ Løfte og bære 12 kg, svarende til en mellem til stor kuffert |
| Do heavy household activities – like scrubbing floors, vacuuming, raking leaves ^a, b^ | Udføre krævende huslige gøremål, så som at vaske gulv, støvsuge eller rive blade sammen |
| Do strenuous activities – like hiking, digging in garden, moving heavy objects, bicycling, aerobic dance activities, and strenuous calisthenics etc. ^a^ | Udføre anstrengende aktiviteter, så som at vandre, grave have, flytte tunge genstande, udføre aerobic og hård træning o.lign. |
|  | |
| Can do (2)  Can do with difficulty or with help (1)  Cannot do (0) | *Kan udføres (2)*  *Kan udføres med besvær eller med hjælp (1)*  *Kan ikke udføres (0)* |

a: adapted from the 5 and/or 6 item scales by Siu, Reuben and Hays;
b: adapted from the Rosow-Breslau Scale ;
c: adapted from the National Health Interview survey

| *^1^ Regarding the walking distance   - If the walking distances can be performed using a walker or a cane the answer is= Can do with difficulty or with help - If a wheelchair is required, the answer is: Cannot d | *^1^ I forhold til gangdistancerne   - Hvis distancen kan udføres ved brug af rollator, stok er svaret= kan udføres med besvær eller hjælp. - Hvis der anvendes en kørestol er svaret = Kan ikke udføres. |
| --- | --- |
