# Supplementary material for: Translation, cross-cultural adaption, validity and reliability of a composite physical function scale for adults aged 65 + years in a Danish context
Source: BMC Geriatr. 2023 Aug 29;23:526. doi: 10.1186/s12877-023-04240-2 (PMC10466833; doi:10.1186/s12877-023-04240-2)
Supplement: Supplementary file 2 — Additional file 2: Supplementary table 2. Distribution of responses from the pretest. [file 12877_2023_4240_MOESM2_ESM.docx]

**Supplementary material 2**

*Supplementary table 2: Distribution of responses from the pretest*

|  | Can do | | Can do with difficulty or with help | | Can not do | | How confident are you that you understood the question correctly (1-10) (n=88) | |
| --- | --- | --- | --- | --- | --- | --- | --- | --- |
|  | n | % | n | % | n | % | mean | (95% CI) |
| Take care of own personal needs – like dressing yourself | 84 | 92 | 8 | 9 | 0 | 0 | 9.98 | (9.9-10.0) |
| Bathe yourself, using tub or shower | 82 | 89 | 10 | 11 | 0 | 0 | 9.86 | (9.7-10.0) |
| Climb up and down a flight of stairs (like to a second story in a house) | 77 | 84 | 7 | 7 | 8 | 9 | 9.85 | (9.6-10.0) |
| Walk outside (one or two blocks) | 82 | 90 | 6 | 6 | 4 | 4 | 9.95 | (9.9-10.0) |
| Do light household chores – like cooking, dusting, washing dishes, sweeping a walkway | 81 | 88 | 3 | 3 | 8 | 9 | 9.97 | (9.9-10.0) |
| Shop for groceries or clothes | 74 | 80 | 9 | 10 | 9 | 10 | 9.92 | (9.8-10.0) |
| Walk ½ mile (6-7 blocks) | 75 | 81 | 8 | 9 | 9 | 10 | 9.93 | (9.9-10.0) |
| Walk 1 mile (12-14 blocks) | 71 | 77 | 10 | 11 | 11 | 12 | 9.91 | (9.8-10.0) |
| Lift and carry 10 lb (full bag of groceries) | 16 | 83 | 3 | 3 | 13 | 14 | 9.97 | (9.9-10.0) |
| Lift and carry 25 lb (medium to large suitcase) | 60 | 66 | 16 | 17 | 16 | 17 | 9.63 | (9.3-9.9) |
| Do heavy household activities – like scrubbing floors, vacuuming, raking leaves | 73 | 79 | 7 | 8 | 12 | 13 | 9.97 | (9.9-10.0) |
| Do strenuous activities – like hiking, digging in the garden, moving heavy objects, bicycling, aerobic dance activities, strenuous calisthenics, etc. | 63 | 68 | 12 | 13 | 17 | 19 | 9.76 | (9.5-10.0) |
